# Added Sugar Consumption in Spanish Children (7–12 y) and Nutrient Density of Foods Contributing to Such Consumption: An Observational Study

**DOI:** 10.3390/nu15030560

**Published:** 2023-01-21

**Authors:** Marta Palma-Morales, María Dolores Mesa-García, Jesús R. Huertas

**Affiliations:** 1 Institute of Nutrition and Food Technology “José Mataix”, Biomedical Research Center, University of Granada, Avda. del Conocimiento s/n., 18071 Granada, Spain; 2 Department of Physiology, Campus de Cartuja s/n, University of Granada, 18071 Granada, Spain; 3 Department of Biochemistry and Molecular Biology II, Pharmacy Faculty, Campus de Cartuja s/n, University of Granada, 18071 Granada, Spain; 4 Institute for Biosanitary Research ibs.GRANADA, 18012 Granada, Spain; 5 Primary Care promotion of Maternal, Child and Women’s Health for Prevention of Adult Chronic Diseases Network (RD21/0012/0008), Institute of Health Carlos III, 28029 Madrid, Spain

**Keywords:** added sugars, childhood obesity, children nutrition, nutritional quality index

## Abstract

Background: Diets rich in free sugars are associated with an increased risk of obesity. The aim of the present study is to estimate the intake of added sugars in the population of Spanish children and analyze the quality of the foods that contribute to this intake. Methods: An observational study was conducted to collect self-reported information on weekly food consumption in 1775 Spanish children (7–12 years). Nutrient contents were obtained from different databases. Two nutritional density indices were constructed taking into account all nutrients provided and compared with two previously described indices. Results: The average consumption of added sugars in Spanish children was 55.7 ± 1.0 g/day. The products that most contributed to added sugar intake were biscuits (13.3%), cocoa powder (11.1%), sweetened yogurts (9.9%), and dairy desserts (8.6%). Among these, dairy products had considerable nutritional value. Parents’ perception of nutritional value was not aligned with the actual nutritional value. Conclusion: The consumption of added sugars was higher than recommended. Public awareness efforts should focus on: (1) the reduction of consumption of low-nutritional quality products containing high amounts of added sugars; (2) the industrial reformulation of most consumed products to reduce sugar content and increase valuable nutrients; and (3) nutritional education.

## 1. Introduction

The prevalence of obesity is rapidly increasing worldwide, with its prevalence having nearly tripled between 1975 and 2016 [1]; thus, obesity constitutes an epidemic that currently affects more than 2 billion people [2]. According to the WHO, in 2016, over 340 million children and adolescents aged 5–19 were overweight or obese [1]. The most recent data in Spain reported a 23.3% and 17.3% prevalence of childhood overweight and obesity, respectively [3]. Obesity is a risk factor for other chronic noncommunicable diseases, such as insulin resistance, dyslipidemia, cardiovascular disease [2,4], dementia, depression, and some cancers [4], which can cause disability and premature death [5] and therefore must be prevented from infancy onward. Global mandates widely support the need for action to promote healthy diets to prevent childhood overweight, including the Sustainable Development Goals [6] and the Rome Declaration on Nutrition [7]. More specifically, the establishment and subsequent recommendations of the Commission on Ending Childhood Obesity considered the prevention of childhood overweight and obesity as an urgent priority [8]. A diet rich in free sugars is associated with an increased risk of obesity, metabolic syndrome, and cardiovascular disease [4]. Within preventive strategies, the WHO [9] recommends reducing the intake of free sugars to less than 10% of total caloric intake, with additional benefits (especially for caries prevention) if consumption is reduced to 5% of total caloric intake [4]. For children, this would mean less than 25 g of free sugars/day. In addition, to maintain a low risk of disease, the EFSA has recommended that the intake of free sugars should be as low as possible in the context of a nutritionally adequate diet [10]. Free sugars include naturally present sugars, i.e., disaccharides and monosaccharides, that are an intrinsic part of foods and beverages, such as honey, syrups, and fruit juices, and therefore cannot be diminished, while added sugars include all monosaccharides and disaccharides added during food processing and culinary preparation [11].

However, although recommendations have been established to reduce these problems, they have not been effective, as confirmed by the increasing rate of obesity and metabolic diseases, especially in children [3]. Hence, it is necessary to establish new, more realistic strategies, taking into account the real food consumption and preferences of children, to establish recommendations to reduce the intake of added sugars. Therefore, the frequency of food consumption should be evaluated, establishing which foods are most consumed and assessing the amount of dietary sugars provided by them. In addition, it is important that these recommendations do not lead to nutritional deficiencies, and other relevant nutrients should not be excluded. Therefore, the aim of the present study is to estimate the intake of added sugars in the population of Spanish children and analyze the quality of the foods that contribute to this intake to establish better recommendations for reducing the intake of free sugars without compromising other relevant nutrients.

## 2. Materials and Methods

### 2.1. Design and Study Population

An observational study was designed in which self-reported information on the weekly food consumption of children was collected through an online survey, as has been done previously in other studies [12,13,14,15,16]. The parents’ perception of the quality of their children’s food was also collected.

The survey was addressed to parents of children aged 7–12 years living in Spain. It was disseminated through social networks to the general population between 1 November and 31 December 2021. Survey participants were parents over 18 years of age, of both sexes, with different levels of education, and residents of all autonomous communities. To calculate the representative sample size, we used the following formula described by Moser and Kalton [17]. To obtain adequate representativeness of the study population (2,603,810 Spanish children aged 7–12 years) with a confidence interval of 95% and a margin of error of 3%, the required sample size was 1068 children. Finally, the survey was answered by 1775 volunteers.

### 2.2. Data Collection Instrument and Study Variables

An online survey was created based on the ALADINO study [5]. The survey targeted adults with healthy children aged 7–12 years who were not on modified diets for any condition or disease. It was completed anonymously and self-administered. The survey consisted of three blocks of questions, organized as follows: (1) respondent profile: age, gender, educational level, and autonomous community; (2) child food consumption: weekly food frequency consumption of foods containing added sugars; and (3) perception of the child’s nutritional habits: questions on the perceived quality of foods that provide added sugars and foods that they would eliminate from their child’s diet (Supplementary Material S1). The weekly food frequency questionnaire was based on question 16 of the ALADINO survey. To detail the frequency of consumption, the ALADINO responses (never, less than once a week, some days, almost every day, every day) were modified to never, less than once a week, once a week, twice a week, three days per week, four days per week, five days per week, six days per week, and every day. In addition, the products of interest in this study (products with added sugars) were added to this question. Another question was also included to determine how many servings were consumed per week. The question was as follows: “On the day your child eats each of the previous foods, how many servings does he or she eat?”. The answers were 1, 2, 3, or 4 or more servings. Combining the answers to both questions resulted in the number of servings per week consumed of each product with added sugars (Supplementary Material S1).

In addition to milk, natural yogurts and packaged juices do not contain added sugars; they are widely consumed by the child population, and there are similar foods that contain added sugars that can be interchangeable. Hence, they have also been included in the questionnaire.

### 2.3. Estimation of the Intake of Added Sugars

The results of the survey provided insight into children’s food consumption patterns. The estimation of the usual portions was assumed using the photographic guide of food portions consumed in Spain [18].

To obtain data on the added sugar content that was not included in labeling, information was requested from different leading food companies. Information was only obtained for plant-based drinks (calculated as the average between soy and almond beverages from one leading brand), infant milks (obtained from one leading brand product), packaged milkshakes (obtained from one leading brand product with 90% milk), and jams (calculated as the average between four different products from one leading brand). For nectars and dressing sauces, the amount of added sugars was estimated as the difference between the sugars declared on the labeling minus the sugars in the zero analogs containing no added sugars. For foods containing lactose (yogurt, dairy desserts, ice cream, and chocolate bars), added sugars were calculated by the difference between total sugars and lactose analyzed by a certified company (SCADA Laboratories S.A.); one leader product in each category was analyzed. In the case of homemade sponge cake, a traditional recipe was considered, and the added sugars were calculated considering those from the sugar and the yogurt. In the case of products that do not have naturally present sugars (packaged pastries, soft drinks, biscuits, breakfast cereals, candies and sweets, cocoa powder, and sports drinks), all sugars were considered to be added.

The Spanish Food Composition Database (BEDCA) [19] was used to determine food composition. For foods considered as a category, e.g., pastries, which encompass several products, an average of the components found in the BEDCA database was assumed. Data not found in the database included the following: plant-based drinks, including soy, oat and almond milks, juices, and nectars, as well as the amount of vitamin D data for packaged milkshakes. Therefore, they were taken from the labeling of an average of at least three different brands present on the market. For the homemade sponge cake, the BEDCA values of the different ingredients were considered. The authors are conscious that some foods included in the analysis (cocoa powder, jams, and dressing sauces) are not consumed as such but as part of different culinary preparations. However, due to the variability of those preparations, these foods are analyzed following their own nutritional labeling.

### 2.4. Calculation of Nutritional Density Indexes

We constructed a nutritional density index per serving (NDIS), reflecting the nutrient density intake per serving of each food, and another daily nutrient intake index (DNII), calculated as a function of the estimated daily amount consumed by children. The NDIS for each product was calculated by adding the ratios between the content of each nutrient per serving and the reference values established by EFSA for the pediatric population [20]. The DNII was calculated by the addition of the ratios between the average daily intake of each nutrient and the reference values established by EFSA for the pediatric population [20]. The reference values for children that have been taken into account are the established average energy requirement (AR) values for total energy; the reference intake (RI) ranges for carbohydrate, fiber, and fat; the recommended dietary intakes (RDI) for protein; the population reference intakes (PRI) for the vitamins and minerals folate, niacin, riboflavin, thiamin, vitamin A, vitamin B6, vitamin C, calcium, iron, and zinc; and the adequate intakes (AI) for α-tocopherol, vitamin B12, and vitamin D, and the minerals magnesium, phosphorus, potassium, and selenium. To take into account the lower bioavailability of tricalcium phosphate added to plant-based drinks and fortified milk, a correction factor of 0.75 was applied for calcium intake from these products, as reported previously [21]. To take into account the lower bioavailability of iron from dairy products and vegetable products, correction factors of 0.15 and 0.1, respectively, were applied as reported elsewhere [22].

$$\mathrm{NDIS}=\sum \frac{\mathrm{Nutrient}\;\mathrm{content}/\mathrm{serving}}{\mathrm{Recommendations}}$$

$$\mathrm{DNII}=\sum \frac{\mathrm{Nutrient}\;\mathrm{contribution}/\mathrm{day}}{\mathrm{Recommendations}}$$

To classify foods, the NDIS was used, considering milk as the reference quality food [23,24], with a calculated NDIS of 3. Accordingly, foods with an NDIS close to that of milk (NDIS > 2.5) were considered to have high nutritional density, foods with an NDIS between 1.5 and 2.5 were considered to have medium nutritional density, and foods with an NDIS less than 1.5 were considered to have low nutritional density. In terms of added sugar content, we considered products with low added sugar content those with less than 5 g per serving, moderate added sugar content those with 5–10 g per serving, high added sugar content those with 10–15 g per serving, and very high added sugar content those with more than 15 g per serving.

Previously proposed food quality indices were also calculated, such as the SAIN (score of nutritional adequacy of individual foods), which considers the content of protein, fiber, iron, calcium, and vitamin C, and the LIM (nutrient to be limited), which considers unhealthy nutrients: sodium, added sugars, and saturated fatty acids [25]. Food with a SAIN index > 5 was considered to be of good nutritional density. A food with a LIM index > 7.50 was considered to be high in unhealthy nutrients.

### 2.5. Statistical Analysis

For the description of the population, categorical variables are presented as percentages and frequencies, and quantitative variables are presented as the mean and standard error of the mean (SEM). For the descriptive analysis of the variables included in the survey, the mean and SEM were calculated using SPSS statistical software.

## 3. Results

### 3.1. Description of the Study Population

The demographic characteristics of the participants are shown in Table 1. Thirty-one percent of the participants were 7 years old, 15.7% were 8 years old, 14.9% were 9 years old, 13.4% were 10 years old, 10.7% were 11 years old, and 14.3% were 12 years old. Regarding the parents’ education, 73.7% had higher education, of which 14% had received education related to health/food sciences (6.3% vocational training and 7.7% graduates), and the rest (26.3%) had elementary or baccalaureate studies. Most participants came from Andalucía (27.1%), Madrid (15.6%), Valencia (12.7%), and Cataluña (11.6%) (Table 1).

### 3.2. Consumption of Added Sugars

**Figure 1:**
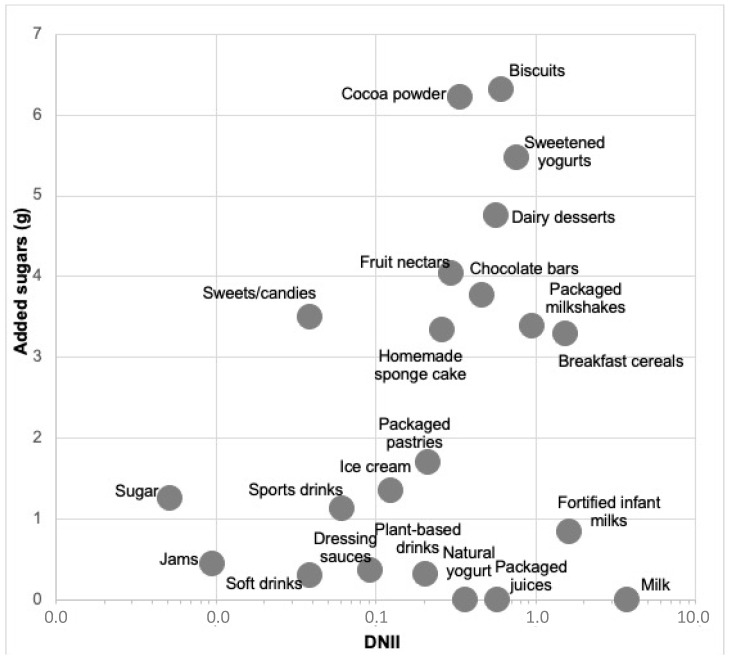
DNII (daily nutrient intake index) vs. added sugars per daily food consumption.

According to the average daily food consumption, the average consumption of added sugars in Spanish children was 55.7 ± 1.0 g/day. Table 2 describes the amount of added sugars provided by each food. Calculations based on food consumption survey data show that the products that provide the highest amount of added sugars to the diet of Spanish children are biscuits (6.3 g/day provided by an intake of 4.3 servings/week), cocoa powder (6.2 g/day provided by an intake of 6 servings/week), sweetened yogurts (5.4 g/day provided by an intake of 3.6 servings/week), and dairy desserts (4.8 g/day provided by an intake of 2 servings/week) (Figure 1, Table 2).

**Figure 2:**
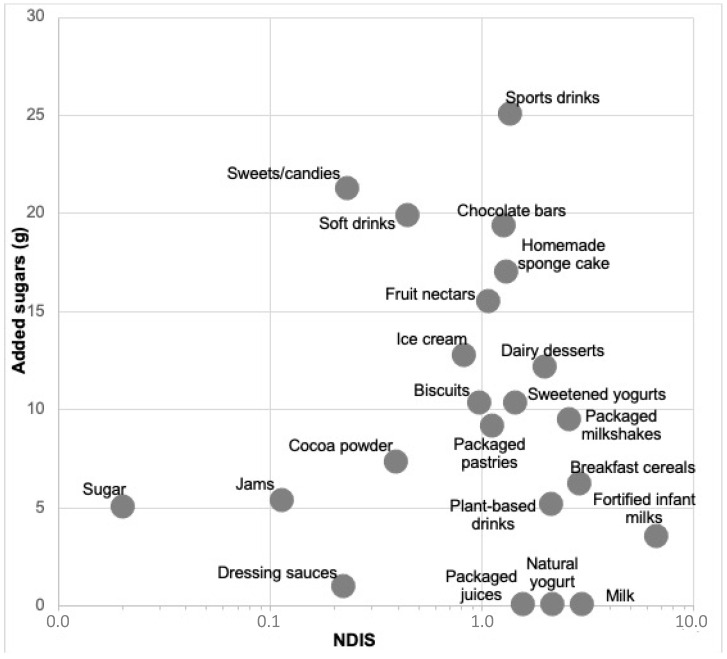
NDIS (nutritional density index per serving) vs. added sugars per serving of food.

The products with the highest amount of added sugars per serving (>15 g/serving) are sports drinks, candies/sweets, soft drinks, chocolate bars, homemade sponge cake, and fruit nectars, which were all considered to be of low nutritional density (NDIS < 1.5) (Figure 2, Table 2). Ice cream, dairy desserts, sweetened yogurts, and biscuits contained from 10–15 g of added sugars per serving (high content), with yogurts and dairy desserts being considered products of medium nutritional density but without considering their beneficial probiotic content (NDIS 1.5–2.5) and ice cream and biscuit products of low nutritional density (NDIS <1.5). Foods with a moderate content of added sugars (5–10 g/serving) included foods with high nutritional density (NDIS > 2.5), such as packaged milkshakes containing 90% milk and breakfast cereals; foods with medium nutritional density (NDIS 1.5–2.5), such as plant-based drinks; and foods with low nutritional density (NDIS < 1.5), such as packaged pastries, cocoa powder, and jams. Finally, foods with low added sugar contents (<5 g/serving) are fortified infant milks (with an NDIS > 3 and very high nutritional density) and dressing sauces (with an NDIS < 0.5 and very poor nutritional density). On the other hand, among the foods with no added sugars considered in this study, packaged juices had an NDIS of 2.19 (medium nutritional density), natural yogurts had an NDIS of 1.59 (medium nutritional density), and milk had an NDIS of 3, indicating a high nutritional density (Figure 2).

### 3.3. Parents’ Quality Perception

In terms of parents’ perceptions of the quality of products containing added sugars, soft drinks, sweets and candies, and packaged pastries were reported to be of very poor or poor quality, while fortified infant milk, plant-based drinks, and homemade sponge cake were considered good or very good quality foods. The remaining food products with added sugars included in the survey were considered to be of normal quality by parents (Table 2).

## 4. Discussion

Currently, both consumers and health care professionals are continuously receiving the message to reduce (or even remove) added sugar consumption, especially from children’s diets, based on the WHO recommendation of less than 10% of total caloric intake [4], which means less than 25 g of free sugars/day [9]. This is justified by scientific studies that show excessive sugar consumption in children, which is considered one of the reasons for the high prevalence of overweight and obesity [4]. However, preventative actions have not been effective, as evidenced by the increased incidence of these diseases. Therefore, other strategies should be attempted. In addition, when reducing added sugars, it is important to take into account the nutritional value of foods so as not to compromise the intake of other nutrients that play a key role in children’s growth and development.

In line with the results found in the ESNUPI [26] and ANIBES [27] studies, which reported mean intakes of added sugars of 38.7 and 48.6 g/day, respectively, our data show an intake of 55.7 g/day of added sugars for children 7–12 years old. Although those studies differ in the methodology used, the results consistently show that the consumption of added sugars is above the recommendations for Spanish children and emphasize the need for strategies to reduce their consumption.

Regarding the main sources of added sugars, the present study shows biscuits, cocoa powder, sweetened yogurts, and dairy desserts as the foods that contribute the most to this intake. These results are in agreement with those from the ESNUPI and ANIBES studies, which reported that added sugars in children’s diets mainly came from yogurts, other sweetened dairy products, bakery/pastry products, chocolates, soft drinks, and sugars/sweets [26,27].

To identify those foods that would contribute the most to reducing added sugars without compromising other nutrients, in the present study, we proposed two nutritional density indices (NDIS and DNII) that take into account a greater number of nutrients not considered by other previously described food quality indices (SAIN and LIM). These indices provide greater objectivity of food nutritional density. However, it is important to take into account that in the present study, the results show the nutritional quality of food as such and not in the final preparation, which obviously would change the perspectives and indices. To validate these new indices, we compared them with previously defined SAIN and LIM indices and verified that the NDIS and DNII data are in line with those obtained by the SAIN and LIM indices. The products with added sugar and the best nutritional density per serving (NDIS > 2.5) are infant milks, breakfast cereals, and milkshakes containing at least 90% milk. These foods contain less than 10 g of added sugars per serving, and they provide less than 4 g/day of added sugars to the total diet.

Milk provides high-value nutrients and has been a basic food since humans developed mechanisms for the persistence of lactase [24], mainly for children. According to the nutritional indices, milk is a high-quality food without added sugars; hence, we have considered it a reference nourishment for the child population with described health benefits [22]. It contains high nutritional quality protein and other naturally present high bioavailable bioactive components, minerals (mainly calcium), and fat-soluble vitamins (mainly vitamins D and A) [23]. Evidence consistently shows that daily cow’s milk consumption improves the intake of energy, protein, vitamins, and minerals [28], hydration, dental and bone health, and growth; furthermore, there is increasing evidence of the effect of cow’s milk on appetite regulation and satiety that may reduce energy intake in overweight or obese children [29,30,31,32,33]. Therefore, it is advisable to promote the consumption of cow’s milk by children to improve their health. These benefits could also be attributed to milkshakes containing more than 90% milk, but taking into account that the food industry is making an effort to decrease added sugars in these products, they cannot be considered a substitute for daily milk itself and should be considered only as a snack alternative in certain situations. As milkshakes are the foods of choice for children, increasing the proportion of milk and reducing added sugars in milkshakes through reformulation policies is a good alternative to improving the nutritional quality of foods intended for children. Indeed, milkshakes are a good source of calcium and vitamin D, which makes them an alternative to meet the nutritional needs of these nutrients, whose current daily intakes are below the average requirement [34].

Among the foods that contribute the most to added sugar intake are flavored and sweetened yogurts. In addition to our NDIS, the DNII indices classify yogurt as a moderately nutritionally dense food, but it should be taken into account that we have considered the serving size (125 g). Indeed, other studies consider yogurt as a food with a high nutritional density that provides the same nutrients as milk [35], although in quantities that may differ slightly. In addition, our indices do not consider that yogurts also contain probiotic microorganisms, whose consumption has an additional beneficial impact on gut microbiota and consequently on health, reducing the risk of chronic diseases such as metabolic syndrome [36], which is a limitation of the nutritional indices. On the other hand, yogurt consumers have been reported to have a higher diet quality than non-consumers [25,35]. However, children mostly consume sweetened yogurts, which provide more than 10 g of added sugars per serving. Therefore, as they are foods of choice for children, it would be advisable to reformulate these products by reducing added sugars.

Consumption of plant-based drinks in children was less than 1 serving/week. These drinks provide vegetable nutrients such as fiber, vitamins, and minerals and are also enriched in some nutrients such as calcium and vitamin D, in an attempt to resemble the composition of milk. However, these nutrients do not reach the same bioavailability and therefore the same nutritional quality as milk [21,22,37,38]. It is therefore important to warn the population that it is not recommended to use plant-based drinks as substitutes for cow’s milk, especially in growing children, as the differences in nutritional composition and bioavailability can lead to deficiencies of certain nutrients, such as calcium and vitamin D, with serious consequences for the child’s development and health [39,40].

The average consumption of pastries is 1.3 servings/week, similar to homemade sponge cakes. According to our data, both types of products are very similar and of low nutritional density (NDIS < 1.5), providing a large amount of added sugars per serving. Differences in the parents’ perception are probably due to the quality of ingredients used for homemade baking compared to other additives used in industrial bakeries. Biscuits were the product of choice for most children (4–5 servings/week), thereby contributing the most to the added sugar consumption of Spanish children. Based on these facts, it is mandatory to encourage the food industry to reformulate this type of food to reduce added sugars and improve its nutritional value.

Cocoa is a food rich in bioactive compounds with beneficial health effects [41,42,43]. However, cocoa targeted at children contains mostly added sugars and provides few essential nutrients, contributing significantly to the daily consumption of those sugars. It is also well considered by parents, who incorporate it into their children’s diets on a daily basis. Although the food industry has formulated new products to improve the nutritional quality of cocoa powder, more effort in research and development of cocoas is needed to ensure a healthier food with acceptable organoleptic characteristics for children.

Regarding sugar-sweetened soft drinks and energy drinks, their negative effect on health is well known due to the high content of added sugars, a high glycemic index, and unhealthy consequences [44,45]. In contrast to the ANIBES study, which shows a consumption of almost 2 servings/week [27], our results showed that Spanish children consumed less than 1 serving/week of sweetened soft drinks. These drinks are being replaced by sugar-free beverages, whose consumption has doubled (0.6 servings/week) compared with data reported by the ANIBES study [27]. The lower consumption of sugar-sweetened soft drinks may be a consequence of the current governmental awareness policies [44], apart from the fact that our data were self-reported, which is a limitation of the present study that may cause some misleading information.

The NDIS classifies juices as products of medium nutritional density and nectars as products of low nutritional density. One limitation of our study is that the survey did not differentiate between juices and nectars. European legislation prohibits the addition of sugars to juices [46], although they can be made from fruit concentrates rich in intrinsic simple sugars. In contrast, nectars may contain up to 20% added sugars, although these legal differences are not well known among consumers. In addition, it is important to alert the population that fruit juices are not comparable to whole fruits [47,48,49,50], as they provide less fiber, vitamins, and minerals than whole fruits, and therefore, they cannot be considered part of the recommended 5 servings/day of fruits and vegetables [9].

Another limitation of the present study is that the food frequency questionnaire (FFQ) was not validated, and the food consumption data were self-reported, based on parental memory and perception, and therefore subjected to bias. In addition, it was not possible to accurately quantify the intake of added sugars since they are neither declared on labeling nor included in the BEDCA database; thus, we only estimated them as previously described. Moreover, no randomization was performed, as the survey was disseminated through social networks to the general population. However, the strength of our study is the large number of participants that were included, exceeding the minimum necessary to be considered representative [17]. In addition, Andalucía was the most reresented region, being also the most populated in Spain [51]. We also consider as a strength the evaluation of not only the sugar content of one food or drink but also the entire nutritional composition by calculating the nutritional density indices.

Our study shows a general picture of the actual current situation of Spanish children’s preferences and nutritional habits related to added sugar consumption, which is the first step toward knowing the key point where clinicians, researchers, and the food industry may join efforts to improve the nutritional profile of the preferred products most consumed by children by reducing added sugars and increasing other essential nutrients, taking into account differences between regions. In parallel, nutrition labeling should be improved, and educational campaigns aimed at both children and parents should be promoted since the perception of parents is not always aligned with the actual nutritional value of food and drinks.

## 5. Conclusions

Spanish children consume more added sugar than is recommended. It is important to educate the population to reduce the consumption of foods containing added sugars without compromising other valuable nutrient intakes. Therefore, in the context of a healthy lifestyle and balanced diet, as long as the intake of added sugars does not exceed the recommended limits, occasional consumption of products with adequate nutritional density could be maintained, prioritizing those with higher nutritional density.

## Figures and Tables

**Table 1:** Characteristics of the participants.

**Age (years)**	**% Population (n)**
7	31.0 (550)
8	15.7 (279)
9	14.9 (264)
10	13.4 (238)
11	10.7 (190)
12	14.3 (254)
**Parent’s level of education**	**% Population (n)**
Primary education	6 (107)
Secondary education	20.3 (360)
Vocational training in other areas	21.8 (387)
Vocational training in health/food sciences	6.3 (111)
Graduates other areas of knowledge	37.9 (672)
Graduates in health/food sciences	7.7 (136)
Others	0.1 (2)
**Autonomous Community**	**% Population (n)**
Andalucía	27.1 (481)
Madrid	15.6 (277)
Comunidad Valenciana	12.7 (225)
Cataluña	11.6 (206)
Castilla y León	6.1 (108)
Castilla la Mancha	5.3 (94)
Aragón	3.8 (68)
Galicia	3.7 (66)
País Vasco	3.3 (59)
Murcia	2.7 (48)
Extremadura	2.2 (39)
Canarias	1.7 (31)
Asturias	1.2 (21)
Cantabria	0.9 (16)
Baleares	0.7 (13)
Navarra	0.7 (13)
La Rioja	0.6 (10)

**Table 2:** Added sugars daily contribution, quality indexes, added sugars, quality perception, and non-consumers of the different products analyzed.

Food	Added Sugars Daily Contribution	DNII	Servings/Week	Added Sugars (g)/Serving	NDIS	SAIN	LIM	Parent’s Perception of Quality	n-NC
Biscuits	6.3	0.61	4.3 ± 0.1	10.3	1.0	3.11	41.08	Normal	158
Cocoa powder	6.2	0.34	6.0 ± 0.1	7.3	0.4	3.21	158.30	Normal	332
Sweetened yogurts	5.5	0.76	3.6 ± 0.1	10.3	1.5	4.95	12.06	Normal	381
Dairy desserts	4.8	0.57	2.0 ± 0.1	12.1	2.0	4.15	14.57	Normal	493
Fruit nectars	4.0	0.3	1.8 ± 0.1	15.5	1.1	22.43	15.56	Normal	798
Chocolate bars	3.8	0.47	2.6 ± 0.1	19.3	1.3	2.80	65.58	Normal	291
Sweets/candies	3.5	0.04	1.2 ± 0.0	21.2	0.2	4.02	142.84	Bad	482
Packaged milkshakes	3.4	0.94	2.5 ± 0.1	9.4	2.6	6.52	7.47	Normal	540
Homemade sponge cake	3.3	0.26	1.4 ± 0.0	17.0	1.3	2.43	35.05	Good	420
Breakfast cereals	3.3	1.55	3.7 ± 0.1	6.2	2.9	9.74	32.08	Normal	516
Packaged pastries	1.7	0.21	1.3 ± 0.1	9.1	1.1	21.78	36.85	Bad	570
Ice cream	1.4	0.12	1.0 ± 0.0	12.7	0.8	3.62	35.96	Normal	506
Table sugar	1.3	0.01	1.8 ± 0.1	5.0	0.0	0.24	400	Normal	1023
Sports drinks	1.1	0.06	0.3 ± 0.0	25.0	1.4	0.26	10.60	Normal	1408
Infant milks	0.8	1.63	1.7 ± 0.1	3.5	6.7	21.78	5.60	Good	1354
Jams	0.4	0.01	0.6 ± 0.0	5.3	0.1	3.75	107.03	Normal	1145
Dressing sauces	0.4	0.09	2.9 ± 0.1	0.9	0.2	10.49	31.31	Normal	187
Plant-based drinks	0.3	0.20	0.4 ± 0.0	5.1	2.2	4.75	5.19	Good	1532
Soft drinks	0.3	0.04	0.6 ± 0.0	19.8	0.5	4.74	24.26	Bad	1076
Packaged juices	0.0	0.57	1.8 ± 0.1	0.0	2.2	30.08	0.16	Normal	798
Milk	0.0	3.71	8.6 ± 0.1	0.0	3.0	9.07	1.78		177
Natural yogurt	0.0	0.36	1.6 ± 0.1	0.0	1.6	9.46	1.63		1041

DNII: Daily Nutrient Intake Index; n-NC number of non-consumers NDIS: nutrient density index per serving; SAIN: score of nutritional adequacy of individuals foods; LIM: nutrient to be limited.

## Data Availability

Not applicable.

## Supplementary Materials

The following supporting information can be downloaded at: https://www.mdpi.com/article/10.3390/nu15030560/s1, Supplementary Material S1: Survey.
