# Supplementary material for: Added Sugar Consumption in Spanish Children (7–12 y) and Nutrient Density of Foods Contributing to Such Consumption: An Observational Study
Source: Nutrients. 2023 Jan 21;15(3):560. doi: 10.3390/nu15030560 (PMC9921831; doi:10.3390/nu15030560)
Supplement: Supplementary file 1 [file nutrients-15-00560-s001.zip › nutrients-2106775-supplementary.pdf]

Added sugar consumption in Spanish children (7-12 y) and nutrient density of foods contributing to such consumption

Marta Palma-Morales[1], María Dolores Mesa-García[1,2,3], Jesús R Huertas[1,4*]

[1]Institute of Nutrition and Food Technology "José Mataix", Biomedical Research Center, University of Granada, Spain. University of Granada, Avda. del Conocimiento s/n. 18016, Armilla, Granada, Spain.

[2]Department of Biochemistry and Molecular Biology II, Pharmacy Faculty, Campus de Cartuja s/n, University of Granada, 18071, Granada, Spain

[3]Institute for Biosanitary Research ibs.GRANADA, 18012 Granada, Spain

[4] Primary care promotion of maternal, child and women's Health for prevention of adult chronic diseases Network (RD21/0012/0008).

[5]Department of Physiology, Pharmacy Faculty, Campus de Cartuja s/n, University of Granada, 18071, Granada, Spain

*Corresponding author: Jesús Francisco Rodríguez Huertas. Institute of Nutrition and Food Technology "José Mataix", Biomedical Research Center, University of Granada, Spain. University of Granada, Avda. del Conocimiento s/n. 18016, Armilla, Granada, Spain. e-mail: jhuertas@ugr.es

I. RESPONDENT PARENT PROFILE

1. Select the age range you belong to:

- 18-24
- 25-35
- 36-49
- 50-65
- Over 65

2. Select your gender:

- Male
- Female

3. What is your level of education?

- Primary education
- Secondary education/Baccalaureate
- Vocational training in health and/or food sciences
- Vocational training in other areas
- University education in health and/or food sciences
- University education in other areas
- Others
- No studies

4. In which Autonomous Community do you currently reside?

- Galicia
- Asturias
- Cantabria
- País Vasco
- Navarra
- La Rioja
- Aragón
- Cataluña
- Valencia
- Murcia
- Andalucía

- Castilla La Mancha
- Extremadura
- Madrid
- Castilla y León
- Islas Baleares
- Islas Canarias
- Ceuta/ Melilla

7. Do you have any children between 7 and 12 years of age?

- Yes
- No

8. Does your child meet any of these criteria?

- No
- Follows a modified diet for any condition or disease
- Has a congenital or metabolic disease
- Has an allergy or intolerance to milk or milk products
- Does not live at home
- Follows a diet prescribed by a physician for pre/post-surgical situation, medical tests, acute or chronic pathologies or certain physiological situations
- Is going through a transitory illness that implies a modification of his/her diet
- Works for or is related to the partner company that is conducting this survey.

II. REGARDING CHILD'S FOOD CONSUMPTION

9. In a typical week, how often does your child eat or drink the following foods or beverages? The question refers to all the intakes during the course of a day: breakfast, mid-morning, lunch, snack and/or dinner.

For each food, the options are:

- Never
- Less than once a week
- Once a week
- Twice a week
- Three days per week
- Four days per week

- Five days per week
- Six days per week
- Every day

Fresh fruit

Vegetables

Natural fruit and/or vegetable juice

Packaged fruit and/or vegetable juices and nectars

Soft drinks with sugar

Sugar-free soft drinks (light, zero or diet)

Breakfast cereals

Cow's milk

Fortified infant milk

Soy, oat, almond drinks

Packaged milkshakes

Cheese

Natural yogurt

Sweetened plain yogurt, flavored yogurt, yogurt with fruit

Dairy desserts (custard, rice pudding...)

Ice cream

Meat

Fish

Salty snacks (potato chips, fried corn, popcorn, peanuts...)

Natural nuts

Candies and/or sweets

Chocolate bars

Cookies

Packaged pastries and cakes (muffins, croissants...)

Homemade sponge cake

Pizzas, French fries, hamburgers, sausages or pies

Packaged fried tomato

Ketchup or other sauces

Eggs

Pasta or rice

White bread

Whole wheat bread

Legumes (lentils, chickpeas...)

Olive oil

Butter

Margarine

Sports drinks (isotonic, energy drinks...)

Sugar

Cocoa powder (to dissolve in milk)

Jam

Packaged sausages

10. On the day your child eats each of the previous foods, how many servings does he/she eat?

For each food, the options are:

- 1 serving
- 2 servings
- 3 servings
- 4 or more

<u>III. ABOUT THE PERCEPTION OF THE CHILD'S NUTRITIONAL HABITS</u>

11. Do you consider the presence of the following nutrients in your child's diet to be....

Insufficient | Adequate | Normal | Excessive | Very Excessive

- Proteins
- Carbohydrates
- Sugars
- Fats
- Vitamins
- Minerals

12. Score the following foods according to your perception of their nutritional quality:

Nutritional quality: Very low | Low | Normal | High | Very high | Very high

Packaged fruit and/or vegetable juices or nectars

Soft drinks with sugar

Breakfast cereals

Fortified infant milk

Soy beverages, oatmeal, almond...

Packaged milkshakes

Sweetened natural yogurt, flavored yogurt, yogurt with fruit...

Dairy desserts (custard, rice pudding...)

Ice cream

Candies and/or sweets

Chocolate bars

Biscuits

Packaged pastries and cakes (muffins, croissants...)

Homemade sponge cake

Packaged fried tomato

Ketchup or other sauces

Sugar

Cocoa powder (to dissolve in milk)

Jam

13. Which of the following foods do you not give or would you like to reduce in your child's diet?

(Multiple choice)

Fresh fruit

Vegetables

Natural fruit and/or vegetable juice

Packaged fruit and/or vegetable juices and nectars

Soft drinks with sugar

Sugar-free soft drinks (light, zero or diet)

Breakfast cereals

Cow's milk

Fortified infant milk

Soy, oat, almond drinks

Packaged milkshakes

Cheese

Natural yogurt

Sweetened plain yogurt, flavored yogurt, yogurt with fruit

Dairy desserts (custard, rice pudding...)

Ice cream

Meat

Fish

Salty snacks (potato chips, fried corn, popcorn, peanuts...)

Natural nuts

Candies and/or sweets

Chocolate bars

Cookies

Packaged pastries and cakes (muffins, croissants...)

Homemade sponge cake

Pizzas, French fries, hamburgers, sausages or pies

Packaged fried tomato

Ketchup or other sauces

Eggs

Pasta or rice

White bread

Whole wheat bread

Legumes (lentils, chickpeas...)

Olive oil

Butter

Margarine

Sports drinks (isotonic, energy drinks...)

Sugar

Cocoa powder (to dissolve in milk)

Jam

Packaged sausages
